# A combination of taurine and caffeine maintains sperm quality in equine semen during chilled storage

**DOI:** 10.5455/javar.2021.h555

**Published:** 2021-11-03

**Authors:** Hermelinda Ramirez-Perez, Hilda Morayma Guerrero-Netro, Paulina Torres-Rodríguez, Maricruz Díaz-Durán, Ana Myriam Boeta-Acosta, Mouhamadou Diaw

**Affiliations:** 1 Depto. de Reproducción, Facultad de Medicina Veterinaria Zootecnia, UNAM, Ciudad de México, México; 2 Instituto de Biotecnología, UNAM, Morelos, México; 3 Department of Clinical Sciences, Faculty of Veterinary Medicine, University of Montreal, Saint-Hyacinthe, QC, Canada; † These two authors contributed equally.

**Keywords:** Equine, chilled semen, caffeine, taurine, motility, viability

## Abstract

**Objective:**

The objective of this study was to evaluate the effects of caffeine and taurine on the motility and viability of chilled equine semen.

**Materials and Methods:**

A total of 12 ejaculates were collected from three mature stallions with proven fertility during the breeding season. The gel-free spermatic fraction of each ejaculate was divided into two aliquots and diluted with a semen extender (either INRA 96® or BotuSemen Gold®). The aliquots were then split and assigned to one of the six treatment groups: control (no supplement), caffeine (2 and 4 mM), taurine (25 and 50 mM), and a combination of caffeine (2 mM) plus taurine (25 mM). Samples were stored at 4°C and analyzed at different time points (0, 24, 48, 72, and 96 h) to evaluate total (TMOT) and progressive (PMOT) motility and viability by computer-assisted sperm analysis.

**Results:**

Regardless of the extender, PMOT and TMOT decreased over time. However, compared with the control, the treatment with 4 mM caffeine significantly mitigated the decrease in PMOT at 72 h. Additionally, semen treated with a combination of caffeine plus taurine maintained a significantly higher PMOT at 96 h, with improved viability at all time points.

**Conclusions:**

The combination of caffeine plus taurine helps maintain chilled equine semen viability and progressive motility up to 96 h independently of the extender used.

## Introduction

Chilled semen is routinely used in equine reproduction. It consists of a suitable extender added to a freshly collected ejaculate, which is cooled slowly and stored between 4°C and 8°C to reduce metabolism and maintain viability over 72 h [1,2]. Fertility rates from chilled equine semen are higher than those from frozen semen, but chilled semen needs to be used over a 2–3-day storage period to maintain high fertility rates [3,4].

During storage, sperm quality deteriorates due to sperm membrane damage and a decrease in motility, resulting in a shorter sperm survival time in the female’s reproductive tract. This deterioration in sperm quality during cold storage may be due to a deficiency in antioxidant defense [5]. The addition of antioxidants to the refrigeration and freezing diluents may be a possible solution to the changes produced in sperm cells. Taurine and caffeine have been previously reported as antioxidants able to enhance the quality of chilled or frozen semen in several mammals [1,6].

The beneficial effects of taurine as an antioxidant in biological systems have been attributed to its ability to stabilize membranes [7] by sequestering reactive oxygen species (ROS) [8] and reducing lipoperoxidation products [9]. Previous studies on Mithun (*Bos frontalis*) indicated that taurine at a dose of 50 mM significantly reduces the percentage of dead spermatozoa [1]. Additionally, in equines, it has been previously reported that the addition of taurine (70 and 100 mM) to INRA 82 extender significantly improved the motility and viability of chilled semen for up to 96 h [10].

Caffeine is a cyclic nucleotide phosphodiesterase inhibitor that stimulates sperm motility, fructolysis, and respiration, and increases cyclic adenosine monophosphate (cAMP) [11]. The effect of caffeine on the semen of domestic animals has been tested for several years. Caffeine has been studied in turkey semen, where a dose of 7.5 mg/ml increased sperm motility [12]. In ram semen, doses of 2 and 4 mM significantly reduce the proportion of dead sperm, increase motility, and maintain it up to 72 h [13]. The effects of caffeine have been studied on post-thaw frozen equine semen. It has been reported that 5 mM can increase progressive motility, but to our knowledge, the effects of caffeine have not been tested on chilled semen [6,14].

The purpose of the present study was to determine the effects of caffeine and taurine, both separately and combined, on motility and viability of chilled equine semen (up to 96 h) to evaluate the efficacy of caffeine and taurine for improving sperm performance in equine reproduction.

## Material and Methods

### Animals and semen collection

The present study was approved by the Subcomité Institucional para el Cuidado y Uso de Animales Experimentales of UNAM (SICUAE.MMVZ-2019 / 1-3). Three animals, of 5–10 years old with a body condition of 3–3.5 on a scale of 1–5 [15], were used in this study. They were housed at the Equine Reproduction Center of UNAM in Tequisquiapan, Queretaro, Mexico (Latitude 20°36'N; Longitude 99°56'W) and maintained under uniform feeding and housing conditions. All stallions had good reproductive characteristics, including normal semen concentration ranges, morphology, total and progressive motility (≥100 million/ml, ≤20%, ≥70%, and ≥75%, respectively). Three ejaculates were collected from each stallion using an artificial vagina (Botupharma) once a week. After collection, the ejaculates were evaluated immediately in the laboratory.

### Semen processing

Initially, semen was evaluated macroscopy and microscopy. Only samples with ≥60% progressive motility and ≤20% abnormalities were processed. The semen was diluted in either INRA 96® (IMV Technologies, L’Aigle,cedex, France) or BotuSemen Gold (Botupharma®) at a final concentration of 25–30 million of spermatozoa per milliliter [16–18]. Aliquots of 1 ml of semen and freezing extender, INRA 96® or BotuSemen Gold, were made and treated with 2 mM caffeine, 4 mM caffeine, 25 mM taurine, 50 mM taurine, and a combination of 2 mM caffeine and 25 mM of taurine, or CTR (control, untreated). Treated semen samples were placed inside an Equitainer® (with a cooling curve of −0.05°C/min) to prevent cold shock for 10 h. Afterward, samples were placed inside a refrigerator at 4°C until 96 h. The treated semen was analyzed for total and progressive motility and viability at 0, 24, 48, 72, and 96 h.

### Semen evaluation

#### Macroscopic evaluation

Macroscopic evaluation included volume, color, and odor. To measure volume and color, the ejaculate was placed in a beaker. Odor was tested to discard the presence of urine or an infection process.

*Morphology*: Morphology was evaluated at the beginning of the experiment for each ejaculate. The sample was diluted 1:1 with PBS and was centrifugated at 300 *g* for 10 min. After the centrifuge, the supernatant was removed. A volume of 0.1 ml was taken from the ejaculate pellet and re-suspended in 0.9 ml of PBS. A slide was made using 10 μl of the sample. The slide was soaked in SpermBlue stain for 45 sec and rinsed with distilled water for 5 sec. The slide was drained at 45°. Once the slide was dried, fixative Eukit was placed in four points of the slide, and a cover glass was placed on top. The slides rested for 5–10 min before the evaluation with the CASA system (SCA® Evolution, version 6.2.0.15, Microptic, Barcelona, Spain), focusing 40x magnification (according to the MICROPTIC standardized test protocol).

*Total and progressive motility:* After incubation with caffeine, taurine, or combining both for different time points, aliquots were warmed up at 37°C in a water bath for 5 min and immediately evaluated.

Total and progressive motility (TMOT and PMOT) were measured using a CASA system with the following parameters: images per second = 25 and square size = 152. Only 10 μl of the sample was placed on a slide, and a cover glass (18 × 18 mm) was placed over the sample. A focus with 10x magnification was used to evaluate five fields for each sample, as previously reported by Wilson-Leedy and Ingermann [19].

*Sperm viability:* The sperm viability test was used to determine if nonmotile sperm were alive or dead. This was carried out by using 18.8 μl of the sample plus 0.2 μl propidium iodide (1 Mm, Invitrogen, Carlsbad, CA, USA) and 1μl Hoechst (1 mM, Molecular Probes, Eugene, OR, USA). After the aliquots were centrifuged at 300 *g* for 5 min. The supernatant was removed, and the resulting pellet was mixed with 10 μl of cold methanol and fixed over a slide. The dry slide was wrapped in aluminum foil and refrigerated at 4°C. The fixed samples were read in the IBT (Cuernavaca, Mexico); each sample was rinsed with distilled water and put over a cover glass. Using a fluorescent microscope with a CASA system, 200 spermatozoa were read for each sample.

### Statistical analyses

All statistical analyses were carried out with JMP software (SAS Institute, Cary, NC). Semen parameters were compared between different treatments by one-way analysis of variance (ANOVA) followed by Duncan’s test. Before analysis, proportional data were transformed to arcsines, and non-normally distributed data were log-transformed. Data are presented as means ± SEM, and *p* < 0.05 was considered statistically significant.

## Results

### Effects on motility

**Figure 1.**
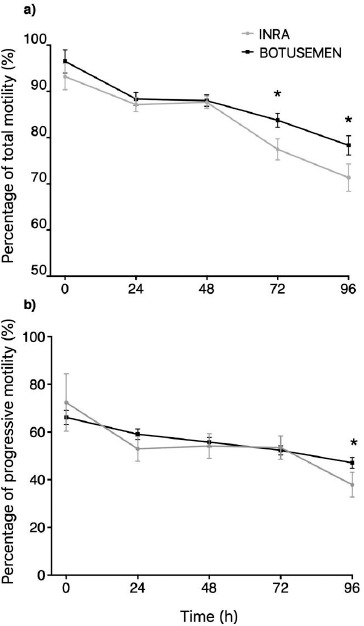
Comparison of total (a) and progressive (b) sperm motility over time, using INRA 96® and BotuSemen Gold® as cooling extenders. * Significant difference between treatments at a certain time point (*p* < 0.05).

Regardless of the treatment group, TMOT, and PMOT decreased over time, as expected. Significantly higher rates of TMOT and PMOT were obtained after 96 h storage with BotuSemen Gold® (78% and 47%, respectively) when compared to INRA 96 (71% and 37.8%, respectively) (Fig. 1).

**Figure 2.**
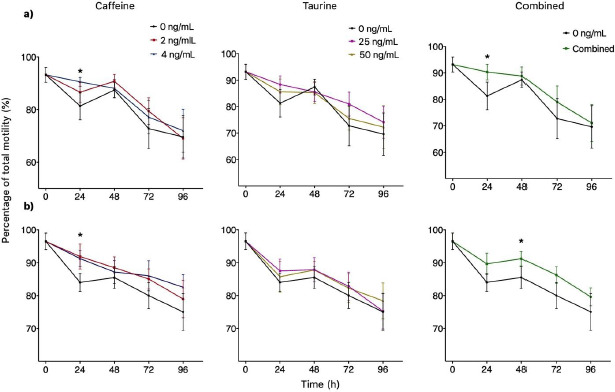
Total motility of spermatozoa over time using INRA 96® (a) and BotuSemen Gold® (b) as extenders. * Significant difference between treatments at a certain time point (*p* < 0.05).

**Figure 3.**
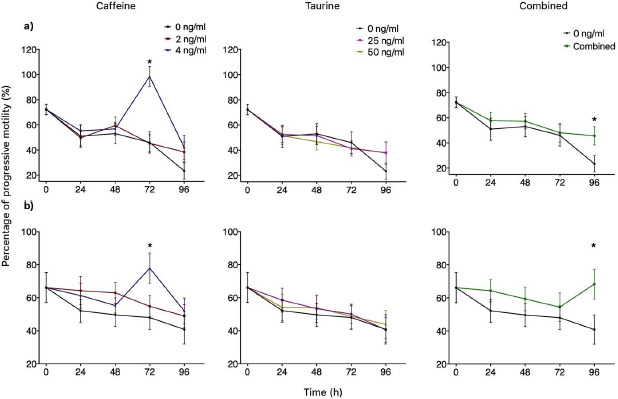
Progressive motility of spermatozoa over time using INRA 96® (a) and BotuSemen Gold® (b) as extenders. * Significant difference between treatments at a certain time point (*p* < 0.05).

Groups treated with either 2 or 4 ng/ml of caffeine significantly increased TMOT at 24 h (*p *< 0.05) when compared to the control independently of the extender used. INRA 96 presented 93% TMOT for both doses of caffeine, while the control was 81%. Similar results were obtained for BotuSemen Gold® where the control group had a TMOT of 84%, while both doses of caffeine had 91% TMOT (Fig. 2). Additionally, 4 mM of caffeine had a significantly higher PMOT for both INRA 96® (77%) and BotuSemen Gold® (86%) at 72 h (*p *<0.05) when compared to the control (72%) (Fig. 3).

The percentage of TMOT at 24 and 48 h from the combined treatment of caffeine plus taurine was not significantly different from time 0 (93%), therefore successfully maintaining the percentage of TMOT for 24 and 48 h for both INRA 96® (90% and 88%, respectively) and BotuSemen Gold® (88% for both times) (Fig. 2). Furthermore, the combination of caffeine and taurine had a significantly higher PMOT at 96 h. INRA 96 presented a PMOT of 45% when treated with caffeine and taurine, while the control had a PMOT of 23%. Similarly, BotuSemen Gold® had 68% PMOT with both caffeine and taurine, when the control had only 40% PMOT (*p *<0.05) (Fig. 3). Taurine did not have significant effects at any given time point for both extenders.

### Effects on viability

**Figure 4.**
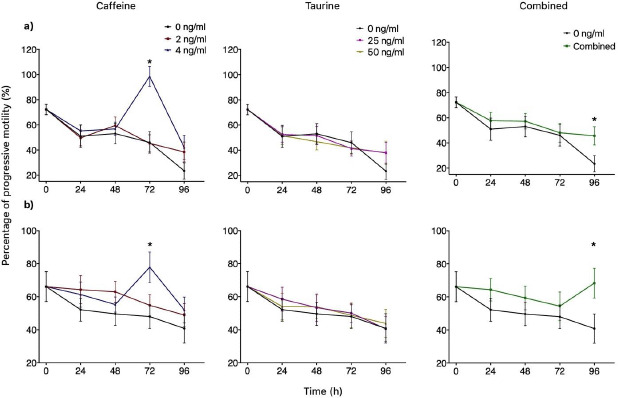
Viability of spermatozoa over time using INRA 96® (a) and BotuSemen Gold® (b) as extenders. * Significant difference between treatments at a certain time (*p* < 0.05).

Semen treated with caffeine at a concentration of 4 mM diluted with INRA 96® (59%) and 96 h (53%) had significantly higher viability when compared to the control (46% and 43%, respectively) (*p *< 0.05) (Fig. 4a). In comparison, semen diluted with BotuSemen Gold® was able to maintain viability with 2 and 4 mM of caffeine at 24 h (68% and 67%, respectively) compared to the control (57%). Additionally, BotuSemen Gold® with 4 mM had a significantly higher cell viability at 72 and 96 h (62% and 60%, respectively) when compared to the control (52% and 46%, respectively) (*p *< 0.05) (Fig. 4b).

A significant increase in viability (*p* < 0.05) was observed at 24 h in comparison to the control (57%) with taurine at a concentration of 50 mM (66%) diluted with BotuSemen Gold®. Furthermore, the same dose diluted with INRA 96 significantly increased viability at 48 h (65%)® when compared to the control (56%) (Fig. 3a).

Finally, the combined treatment of caffeine plus taurine significantly maintained the viability starting at 24 h (67%) and up to 96 h (66%) with BotuSemen Gold®, while INRA 96® kept the viability higher starting at 48 h (65%) until 96 h (57%) when compared to the control (43%) (*p *< 0.05) (Fig. 4).

## Discussion

To our knowledge, this is the first study to prove that a combination of taurine plus caffeine significantly maintained the viability and total and progressive motility of equine chilled semen up to 96 h independently of the extender used.

Equine chilled semen is able to maintain the sperm quality up to 24–48 h during storage depending on the breed, as previously reported [20]. During storage, there is a significant increase in DNA defragmentation and a decrease in the percentage of viability [20,21]. Therefore, different studies attempt to increase the time of chilled semen storage by adding antioxidants. In this study, total and progressive motility decreased over time, but BotuSemen Gold® reduced the motility to a lower percentage compared to INRA 96®. A possible reason for this gradual reduction in percentage might be due to the presence of cholesterol in BotuSemen Gold®. Cholesterol replaces phospholipids in the spermatic membrane, avoiding premature sperm capacitation and protecting the sperm membrane [22,23].

Our results showed that concentrations of 2 and 4 mM of caffeine included in cooling extenders might significantly maintain the total motility in equine semen until 24 h of storage and maintain progressive motility until 72 h using 4 mM of caffeine. Similarly, a positive effect was reported with 4mM of caffeine on total and progressive motility in chilled ram semen until 72 h [13]. The effects of caffeine over motility might be due to the positive effect of the inhibition of phosphodiesterase action, which results in an increase of intracellular cAMP used by the spermatozoa as energy to increase sperm motility [24]. Additionally, a long effect on sperm motility in buffalo chilled semen until 216 h (9 days) has been reported using a higher concentration of caffeine (7 mM) [12].

In our study, concentrations of 2 and 4 mM of caffeine significantly maintained sperm viability at 24 h. In addition, 4 mM of caffeine maintained sperm viability until 72 and 96 h during chilled storage. Viability is related to plasma membrane integrity; it has been previously reported that caffeine reduced sperm apoptosis by maintaining the integrity of the plasma membrane in chilled semen [13].

Taurine at a concentration of 50 mM demonstrated that it is capable of maintaining sperm viability during cool storage at 24 and 48 h. Additionally, 50 mM of taurine significantly improved the percentage of sperm viability and intact plasma membrane in bull semen [1]. Similar results were obtained using 25 mM of taurine in ram semen at 72 h of storage [25]. Taurine helps maintain the acrosome’s integrity, stabilizes the spermatic membrane, and increases motility [26]. Taurine can degrade ROS and protect cells against oxidative stress, resulting in the maintenance of sperm viability [5].

The most marked effect was observed with the combined treatment of caffeine plus taurine, which positively affected sperm motility and viability at all time points (24, 48, 72, and 96 h). The effects of caffeine and taurine on sperm cells have been studied separately, but to our knowledge, no previous studies have investigated their combined effects in equine chilled semen. Therefore, the combined mode of action remains unclear. We suspect that ROS formation occurs naturally over time during storage and compromises the integrity of sperm, which is mitigated by taurine, allowing caffeine to take effect on the motility of healthier sperm cells [27]. However, further studies are required to determine the effects of caffeine and taurine on sperm fertilization rates and, consequently, pregnancy rates.

## Conclusion

Evidence suggests that caffeine combined with taurine might improve the characteristics of chilled equine semen up to 96 h. However, their optimal concentrations must be determined before clinical use. Future studies should also focus on investigating the effects of these substances on fertility when they are added to chilled semen.

## List of abbreviations

ANOVA, one-way analysis of variance; cAMP, cyclic adenosine monophosphate; PMOT, progressive motility; TMOT, total motility; ROS, reactive oxygen species; min, minute; sec, second; h, hour.
